# Changes in the microflora on the seed surface and seed vigor of maize (*Zea mays*) under different conditions

**DOI:** 10.1371/journal.pone.0311258

**Published:** 2024-11-21

**Authors:** Junming Zhang, Zhenzhen Xing, Fengxu Gu, Yulu Wang, Tianbo Wang, Junying Chen

**Affiliations:** Agronomy College of Henan Agricultural University, Zhengzhou, China; University of Georgia, UNITED STATES OF AMERICA

## Abstract

Seed vigor encompasses the germination capacity, ability to form seedlings, and potential for production of seeds, and during storage, the deterioration of seed vigor is an inevitable biological process. However, changes in the microflora of the seed surface and seed vigor under different storage conditions have rarely been studied. In this study, the changes in fungal species on the surface and embryo and their effects of the hybrid maize cultivar Zhengdan958 seeds under different storage conditions were studied. The seed vigor was evaluated according to standard germination, MDA content, respiration rate, ATP content and the integrity of the ATP synthase subunits of seed embryos, with the aim of providing a basis for revealing the molecular mechanism of seed deterioration. The results revealed that at 33% relative humidity (RH), the dominant microflora constituent on the seed surface was *Fusarium* sp. In the seed embryo, the dominant microflora constituent was *Aspergillus fumigatus*. At 91% RH, the dominant microflora constituent on the seed surface was *Aspergillus Jensen*. In the seed embryo, the dominant microflora constituent was *Penicillium* sp. With the increased RH in the storage environment, the seed germination rate decreased by 86.67%. The respiration rate decreased by 0.04 mg·g^-1^·h^-1^ after 24 h imbibition. The seed embryo was hardly stained via TTC. The MDA content increased by 0.99 nmol·g^-1^, and the ATP content decreased by 0.33 μmol·g^-1^ after 24 h imbibition. The mRNA integrity of ATP synthase α, β, γ and δ subunits, except for ε subunit, in the seed embryo decreased to different degrees. These findings suggest that a change in the microflora is one of the most important factors causing a decrease in or total loss of seed vigor.

## Introduction

Seeds are the keystones of agriculture, and high-quality seeds are crucial for ensuring high crop yields [1]. The vigor of the seeds stored under different conditions significantly differs. The reasons for this include genetic factors of seeds [2] and environmental factors, especially the microorganisms around or on the seeds [3].

Different microbial communities colonize the surface and interior of plant seeds [4], among which the most abundant species are bacteria, followed by molds, actinomycetes and yeasts, which are less abundant. Although many bacteria are present in seeds, their effects on the safety of stored seeds are limited compared with those of fungi, such as molds [5]. Mominzai et al. [6] investigated 11 different regions in Afghanistan (Baghlan Province) and reported that 13 species of fungi were detected in 20 wheat samples. Among them, *Aspergillus flavus*, *Aspergillus niger*, *Penicillium* spp., *Cladosporium cladosporioides*, and *Acremonium* spp. were the dominant microflora on the surface of wheat. Riba et al. [7] studied parasitic fungi in wheat in Algeria and reported that the average number of fungi in 85 wheat samples ranged from 275 cfu·g^-1^–277 cfu·g^-1^, in which *Aspergillus flavus* and *Aspergillus* were the dominant microflora constituents. Lacey [8] reported that wheat stored in underground warehouses in southeastern Iran was contaminated mainly by *Aspergillus flavus*.

The effects of parasitic fungi on seeds are manifold. The fungal pathogens can inhabit both the external and internal parts of the seed, including the outer surface and inner embryo, leading to discoloration and a decrease in seed quality [9]. When the seed is cracked or the seed coat is penetrated, the fungus parasitizing the seed surface will quickly invade and propagate and produce toxic metabolites, thus decreasing seed vigor and impacting the morphogenesis of seedlings [10]. Kuang [11] reported that 5 genera and 8 species of fungi had different effects on the germination and growth of three major barley cultivars in Yunnan; among them, *Fusarium flocciferum*, *Fusarium tricinctum* and *Fusarium avenaceum* had obvious inhibitory effects on seed germination, root formation and plant growth, and most seedlings experienced necrosis after germination. Srivastava et al. [12] inoculated seeds with seed-borne fungi, such as *Aspergillus flavus*, *Aspergillus niger* and *Fusarium*, and the seed germination rate and seed vigor index decreased significantly. *Aspergillus flavus* produced aflatoxins that inhibited seed germination, and *Fusarium* had an obvious inhibitory effect on seedling growth.

Seeds are affected by the temperature and relative humidity (RH) of the external environment during storage [13]. A change in seed moisture content increases the activity of microorganisms in the seed sphere [14, 15].

Al-Askar et al. [16] revealed that alterations in relative humidity and temperature were the primary factors influencing the growth, survival, and dissemination of fungi species. Robertson et al. [17] also reported that fungal invasion and a decrease in the germination rate were positively correlated with increases in the seed water content and storage time.Under the same temperature conditions, when the water activity is high, the microflora on the surface of the seeds will exhibit rapid colony production and mycelial growth [18]. After contact with the seed, these fungal mycelia enter the suspensor region through micropores, infect the embryo. These results in excessive accumulation of mycotoxins, and then reduce the seed quality [19]. Zhengdan958 used to be a hybrid maize cultivar with the highest planted area in China. However, changes in parasitic fungi on the seed surface and seed vigor under different storage conditions have not yet been reported. Understanding these changes would provide theoretical support for the conservation of germplasm resources.

In this work, to investigate the correlation between changes in seed surface flora and viability under different storage conditions, we conducted an analysis of the changes in the microflora on the seed surface of the maize cultivar Zhengdan958 under different storage conditions via internal transcribed spacer (ITS) sequence analysis. Additionally, we examined seed vigor according to the standard germination, MDA content, respiration rate, ATP content and integrity of the ATP synthase subunits of seed embryos. These findings provide a basis for further revealing the mechanism of seed senescence.

## Materials and methods

### Material sources and storage conditions

The seeds of the hybrid maize cultivar Zhengdan958 used in this study were produced at the seed production base in Zhangye County in Gansu Province, China. The seed storage conditions were set according to those reported in Jayas and Mazza [20]. Briefly, the seeds were suspended in a sealed container for 2 months (60 d) at room temperature 26±2°C, with RHs of 33% and 91%. Experimental research and field studies on plants (either cultivated or wild), including the collection of plant material, were conducted in compliance with relevant institutional, national, and international guidelines and legislation.

### Calculation of the seed surface area and seed embryo area

Seeds of 30 maize seed were randomly selected, and three biological replicates were set up. The seed surface area was determined via Eq (1), as previously reported by Nemenyi and Szodfridt [21]. The seed embryo area was calculated via the segmentation method, and the seed embryo was transformed into a rectangle, which was determined via Eq (2):

S=−177.8+17.1614a+8.60176b+26.8866c (1)

S: surface area (mm^2^); a, b, c: grain length, width, and thickness (mm), respectively

S=a×b (2)

a, b: Length of two adjacent sides (cm).

### Morphological identification of fungi on the seed surface and on the embryo

The distribution of fungi on the seed surface and on the embryo was observed via an Anyty3R-CLSTM01 stereoscopic microscope via methods described in the books *Taxonomy of Fungi* [22] and *Plant Pathogenic Mycology* [23]. The fungus species were preliminarily identified according to the color, appearance, and size of the conidia and colonies.

### Identification of fungi isolated from the seed surface via molecular biology methods

#### Isolation of the fungi

The procedure for the isolation of fungi from the seed surface and embryo used in this work was described previously by Xing et al. [10]. The dilution coating method was used for strain isolation. A random sample of approximately 1 g was taken, and the bacteria were washed with distilled water from the whole seed and seed embryo parts. The prepared bacterial solution was sequentially diluted into suspensions of 10^−2^, 10^−3^, 10^−4^, 10^−5^, 10^−6^ and 10^−7^ gradients. From each of these dilutions, 0.4 ml was pipetted into potato medium (PDA) with a sterile pipette gun and spread evenly with a spreading stick. Three replicates were made for each dilution, and another blank was used as a control. The strains were inverted and cultured in a constant temperature incubator at 28°C, and the growth of the strains was observed after 3 days. The number of fungi at each dilution and their corresponding colony morphology were recorded via observation under a magnifying glass.

#### Purification of the fungi

The strains were purified via the liquid medium inoculation method. When the isolated strains entered the growth period, the colony morphology was observed, and the spores of individual strains were picked with a sterile gun tip in liquid medium and placed in an incubator at 28°C for overnight cultivation. The culture was continued on a new medium via the plate spreading method. The above steps were repeated until the strain obtained was pure.

#### Molecular identification of strains

The fungal DNA was extracted via an Ezup column fungal genomic DNA extraction kit and detected via 1% agarose gel electrophoresis. The fungal internal transcribed spacer (ITS) region universal primers ITS-L: 5’-CTTGGTCATTTAGAGGAAGTAA-3’ and ITS-L: 5’-TCCTCCGCTTATTGATATGC-3’ were used for Polymerase Chain Reaction (PCR) amplification. The amplification procedure was as follows: total reaction volume 50 μl; primer ITS-F and ITS-R 2 μl; DNA template 5 μl; ddH_2_O, 16 μl; and 2×EasyTaq enzyme, 25 μl. The reaction procedure was as follows: 94°C at 5 min for predenaturation; 34 cycles of 94°C for 30 sec for denaturation; 48°C for 30 sec for annealing; and 72°C for 30 sec for extension; 72°C, extension for 5 min; and 4°C for preservation. The PCR products were observed by 1% agarose gel electrophoresis with a voltage of 150 V and a current of 100 mA for 20 minutes.

The PCR products (500–750 bp) were purified via a SanPre column PCR product purification kit and sequenced at Shenggong Biotech (Shanghai) Co., Ltd. The sequence data were aligned with the GenBank database in NCBI to identify the fungal strains.

### Evaluation of maize seed vigor

#### Germination assay

A germination assay was carried out according to the modified Chen method [24]. Briefly, 30 seeds were selected and sown on two pieces of moistened paper, with 7 biological repetitions per treatment. A seed was considered germinated when its radicle protruded from the seed coat and reached 1–2 mm in the dark at 26±2°C. After 12 h of imbibition, the number of germinated seeds was counted every 2 h until 48 h. The germination rate was determined via Eq (3):

Germinationrate=(G/N)×100% (3)

G, Number of germinated seeds. N, Total number of seeds tested.

*Determination of seed viability*. The cell viability was determined via TTC staining as described by Ciacka et al. [25] followed with some modifications. Briefly, place the seeds under different storage conditions in clean petri dishes and use a scalpel to cut longitudinally along the central axis to ensure the integrity of the tissues in the embryo. Soak the cut seeds in 0.05% TTC solution (buffer: Tris-HCl, pH7.4). Stain at 30°C for 45 min. After washing with distilled water several times, wipe dry and take pictures.

*Determination of malondialdehyde content in seed embryos*. The content of MDA was measured via thiobarbituric acid-reactive substances (TBARS) according to the methods of Latef and Tran [26] with some modifications. Briefly, About 20 g of seed embryo at different relative humidity was randomly separated, with 12 biological repetitions per treatment, homogenized with 10% trichloroacetic acid and centrifuged at 8000×g for 20 min. Two milliliters of extract was mixed with 2 mL of 0.6% TBA and then incubated for 30 min in a boiling water bath. The absorbance was measured at 532 nm, 600 nm and 450 nm with a spectrophotometer. The MDA content was determined via Eqs (4) and (5):

MDAconcentration(μmol/L)=6.45×(OD532‐OD600)‐0.56×OD450 (4)

MDAcontent(μmol/g)=c×v/w (5)

c, v, w: MDA concentration, total volume of extract, and fresh weight of material, respectively.

*Determination of the seed respiration rate*. The seed respiration rate was determined according to the method described by Zhang and Yan [27] with some modifications. Randomly weighed about 20 g of seeds from different treatments with 12 biological repetitions per treatment and performed acid‒base titration via the small basket method. The experimental group without seeds was used as a blank control, and the amount of oxalic acid (V1) was determined until the solution changed from pink to colorless. The blank control was titrated, and the amount of oxalic acid (V0) was determined. Seeds that had been allowed to swell for 24 h were randomly weighed to 20 g. The experimental method was the same as above.


Respirationrate(mg/(g·h))=(V0‐V1)/reactiontime(h)/seedweight(g)

*Determination of ATP synthetase content*. A seed embryo ATP content assay kit (AKOP004U, Boxbio) was used according to the manufacturer’s instructions.

*Evaluation of the mRNA integrity of the ATP synthase subunit*. The mRNA integrity of ATP synthase subunits in seed embryos was evaluated via the double primer blocking method described by Wang et al. [28]. The value calculation method was as follows: R = 2 ^-ΔCtx^/2 ^-ΔCt0^, Δ Ctx = experimental group Ct (5’ end)—experimental Ct (3’ end), and Δ Ct0 = controls (5’ end)—control Ct (3’ end). The more severe the oxidative damage to mRNA is, the larger the ΔCt value and the smaller the corresponding R value.

### Statistical analysis

The data are expressed as the means ± standard deviations, and the statistical significance of differences between two groups was assessed using Student’s t-test, conducted in Excel 2020 (Microsoft USA), SPSS 26.0(IBM、USA) and Graphpad Prism 10 (Microsoft USA). Differences between two groups, the asterisks represent a statistically significant difference at * for P≤0.05, ** for P≤0.01, *** for p< 0.001, **** for p< 0.0001 and ns (no significance), were considered statistically significant.

## Results

### Observation of the microflora on the seed surface under different storage conditions

**Fig 1:**
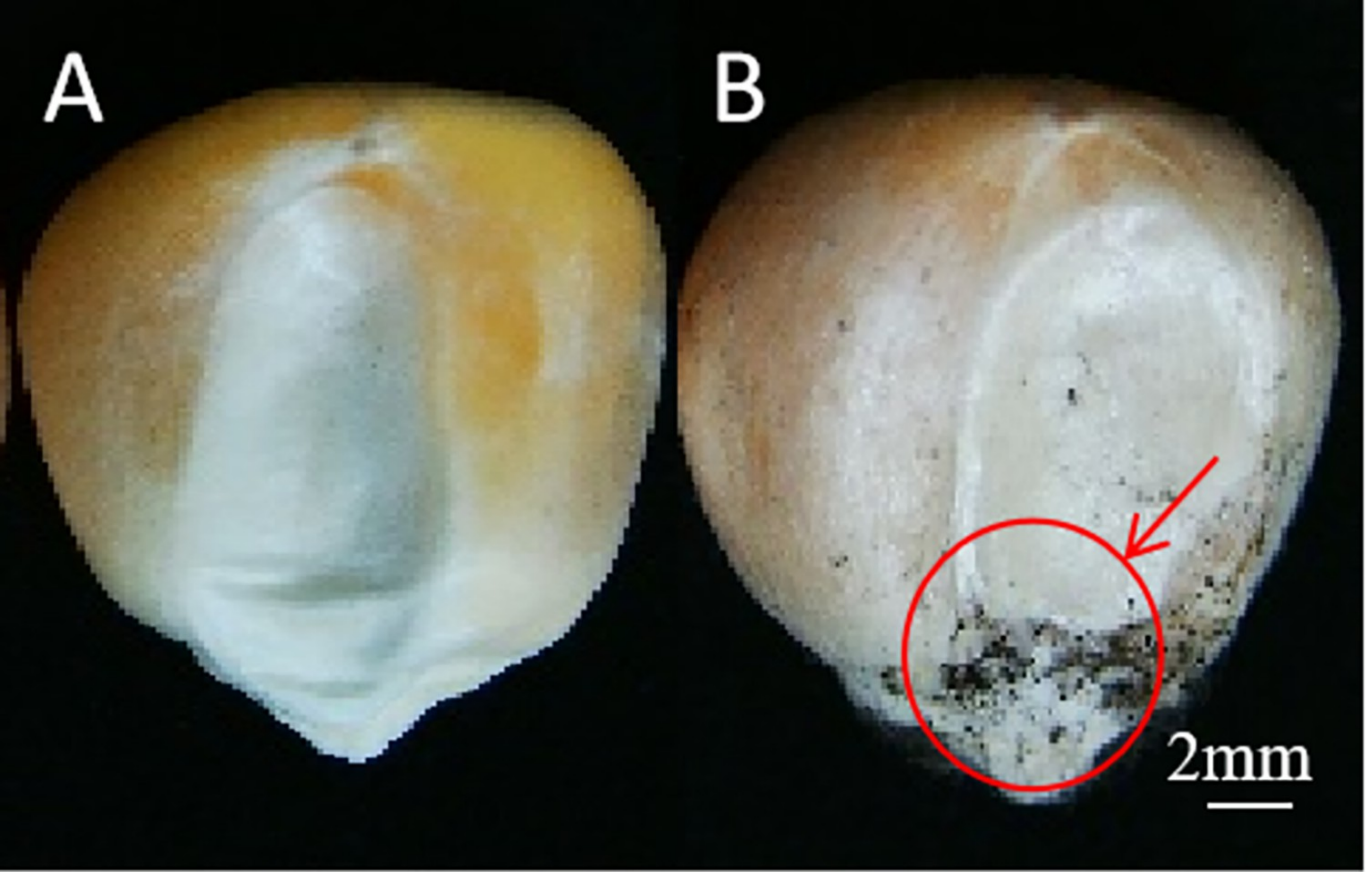
Distribution of fungi on the surface of seeds stored under different conditions. A and B represent seeds stored for 60 d at 33% RH and 91% RH, respectively.Scale bars: 2 mm.

The distribution of the microflora on the seeds stored for 60 d under different storage conditions was observed via stereomicroscopy (Fig 1). The results revealed no obvious mildew on the seeds stored for 60 d at 33% RH. However, there was discoloration and evident mildew on the surface of the seeds stored for 60 d at 91% RH (Fig 1B), especially at the base of the seed embryo.

### Changes in fungi on the surface of seeds stored under different conditions

**Fig 2:**
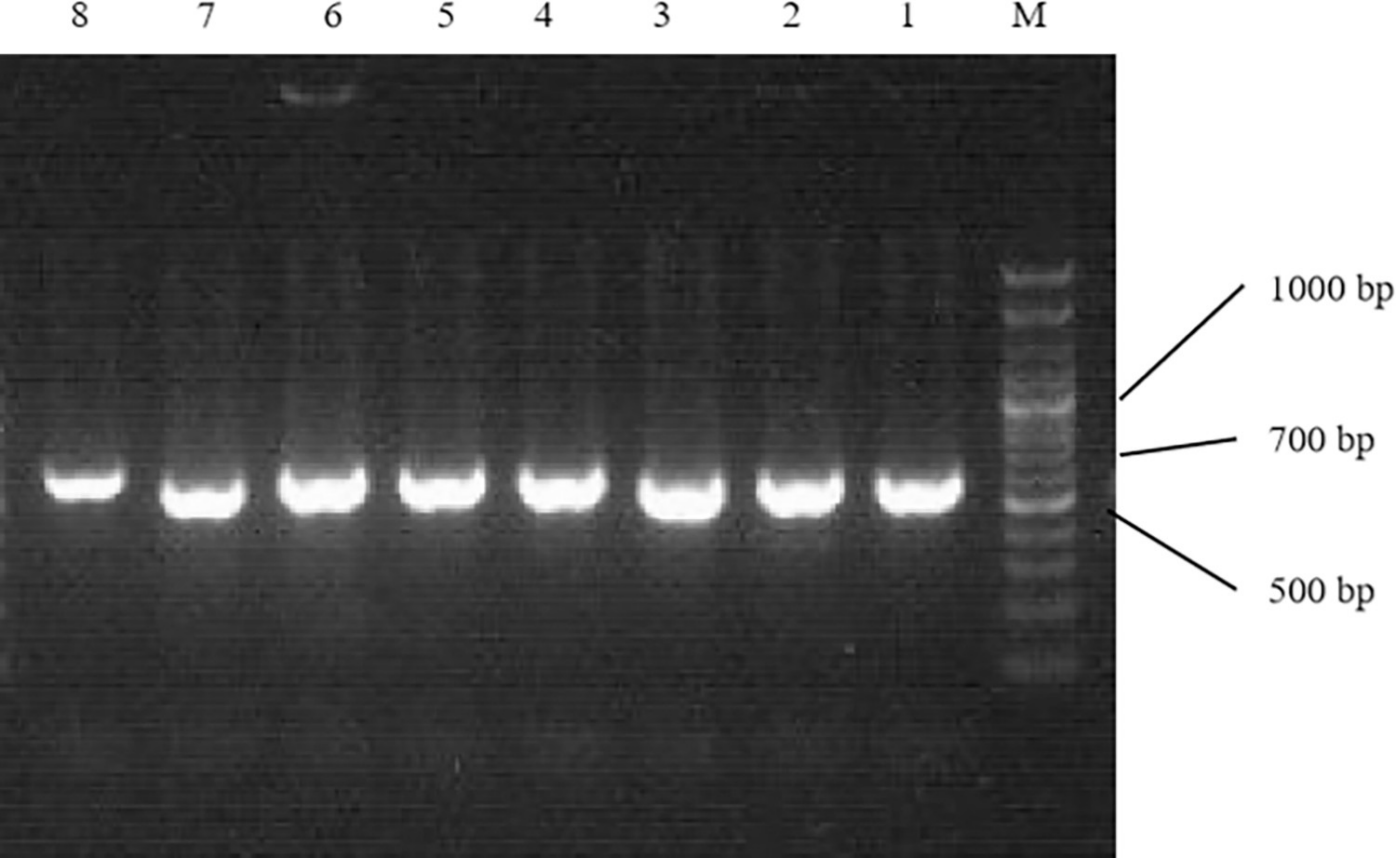
Electrophoresis pattern of fungal DNA PCR products with universal primers on seed samples. M: 100–3000 bp Ladder-K. 1–6: Strain sample number.

The DNA of fungal samples isolated and purified from the seed surface and embryo under different storage conditions (33% RH and 91% RH) was detected via PCR with the universal primers ITS-L: 5’-CTTGGTCATTTAGAGGAAGTAA-3’ and ITS-L: 5’-TCCTCCGCTTATTGATATGC-3’. The results of PCR amplification revealed clear bands (Fig 2), indicating that the extracted DNA was high quality and could be used for sequencing and fungal identification (Table 1). The results of identification are shown in Table 1.

**Table 1:** Comparison of fungi present on the seed surface and embryos of Zhengdan958 under different storage conditions.

Microflora species	Characteristics of fungi	Colony count (cfu/cm^2^)	Proportion(%)
33%RH: Zhengdan958 entire seed surface			
Fusarium	The colony was white and velvety with a branching and septating mycelium	43	69.54
*Aspergillus fumigatus*	The colony was fluffy-shaped with spherical and spiny conidia	26	32.91
Sarocladium	The colony was soft and wrinkled with a fibrous mycelium	3	3.8
Rhizomucor	The mycelium was septate and multinucleate with arcuating branches	2	2.53
Aspergillus flavus	The colony was a grayish green color with a intricat mycelium	2	2.53
Talaromyces funiculosus	The colony was powdery and slightly raised with a transversing septal mycelium	1	1.27
Aspergillus niger	The colony was dark brown with a well-developed and branched mycelium	1	1.27
Cladosporium tenuissimum	The colony was dark brown with a cylindrical septal mycelium	1	1.27
		79	100
33%RH: Zhengdan958 embryo surface			
*Aspergillus fumigatus*	The colony was fluffy-shaped with a spherical and spiny conidium	167	39.95
Aspergillus flavus	The colony was grayish green with a complex mycelium	139	33.25
Talaromyces funiculosus	The colony was powdery and slightly raised with a transversing septa’s mycelium	28	6.7
Aspergillus niger	The colony was dark brown with the well developed and branched mycelium	28	6.7
Cladosporium tenuissimum	The colony was dark brown with the cylindrical septum’s mycelium	28	6.7
Fusarium	The colony was white and velvety with branching and septating mycelium	28	6.7
		418	100
91%RH: Zhengdan958 entire seed surface			
Aspergillus jensenii	The colony was dark green with a bulbous mycelium ends	60×10^4^	56.6
Aspergillu sversicolor	The colony was diverse in color with radiating conidia	22×10^4^	20.75
Penicillium	The colony was gray-green, with a multicelluar branching mycelium	14×10^4^	13.21
Aspergillus aureus	The colony was dark golden velvet with a well-developed and branched mycelium	3×10^4^	2.83
Penicillium citrinum	The colony was dark golden velvet with a well-developed and branched mycelium.	1×10^4^	1
Fusarium	The colony was white velvet with a branching and septate mycelium	2×10^4^	1.89
Aspergillus fumigatus	The colony was fluffy-shaped,with the spherical and spiny conidium	4×10^4^	3.77
		106×10^4^	100
91%RH: Zhengdan958 embryo surface			
*Penicillium*	The colony grayish green antler with a multicellullar branching mycelium	111×10^4^	79.86
*Aspergillus* fumigatus	The colony was fluffy-shaped with a spherical and spiny conidium	28×10^4^	20.14
		139×10^4^	100

Note: The whole areas of the seed surface and the area embryo were approximately 1.89 cm^2^ and 0.24 cm^2^, respectively.

**Fig 3:**
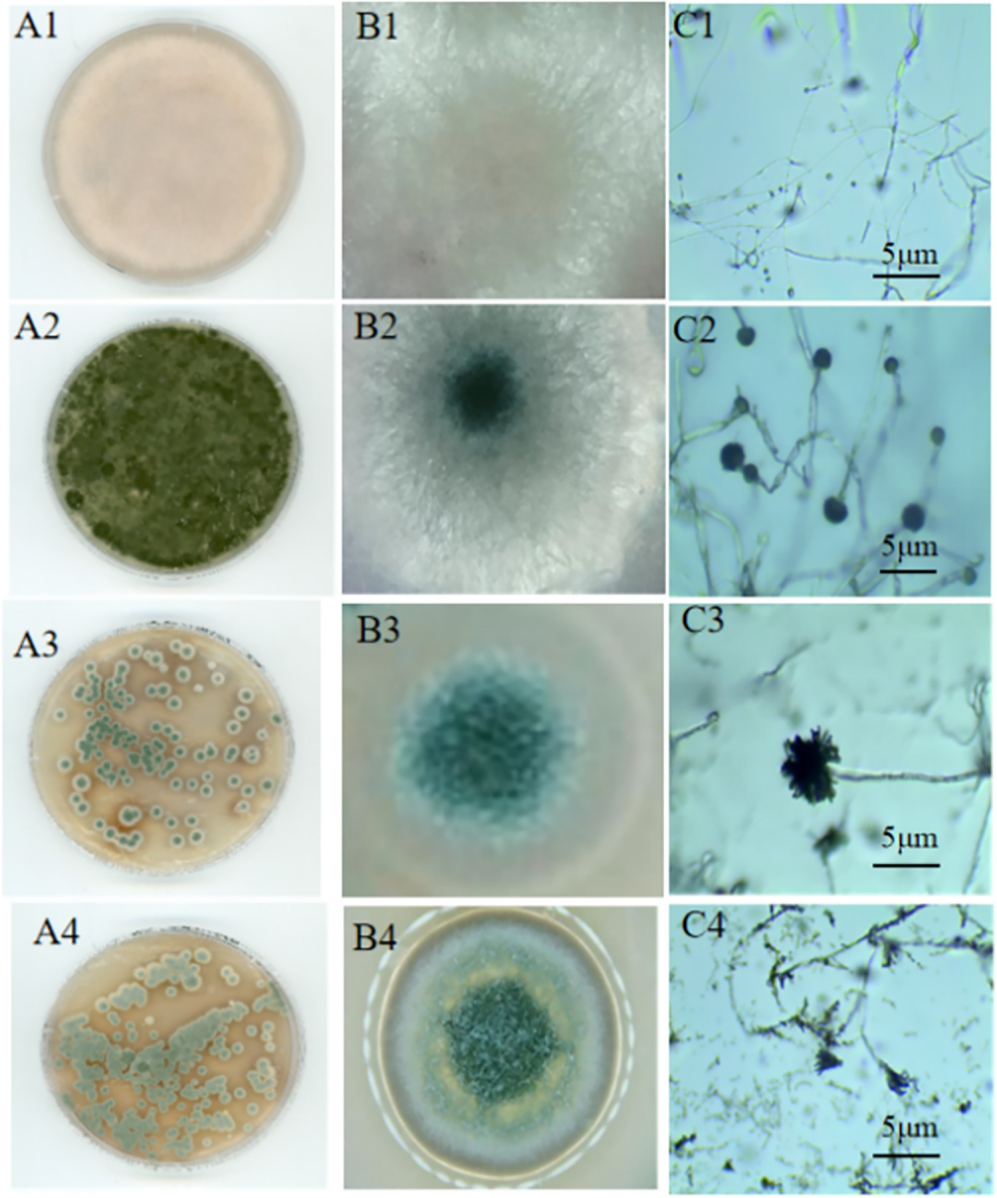
Colony morphology of the predominant microflora constituents. A1, B1, and C1 represent the cultured colonies and mycelia of *Fusarium* and microscopy images, respectively; A2, B2, and C2 represent the cultured colonies and mycelia of *Aspergillus fumigatus* and microscopy images, respectively; A3, B3, and C3 represent the cultured colonies and mycelia of *Aspergillus jensenii* and microscopy images, respectively; A4, B4, and C4 represent the cultured colonies and mycelia of *Penicillium* and microscopy images, respectively. Scale bars: 5 μm.

A total of 8 species of fungus were identified from the seeds stored for 60 d at 33% RH, and the total colony number was 79 cfu·cm^-2^. The dominant microflora constituent on the seed surface was *Fusarium* (relative abundance: 69.54%) at a density of 43 cfu·cm^-2^ (Fig 3, A1), followed by *Aspergillus fumigatus* (relative abundance: 32.91%), with a microflora density was 26 cfu·cm^-2^. The remaining species were *Sarocladium* sp., *Rhizomucor*, *Aspergillus flavu*s, *Talaromyces funiculosus*, *Aspergillus niger*, and *Cladosporium tenuissimum*. In the seed embryo, a total of 6 species were identified, and the total colony number was 418 cfu·cm^-2^. The dominant microflora constituent was *Aspergillus fumigatus* (relative abundance: 39.95%) at a density of 167 cfu·cm^-2^ (Fig 3, A2), followed by *Aspergillus flavus* (relative abundance: 33.25%) at a density of 139 cfu·cm^-2^. The same colony number was identified for *Talaromyces funiculosus*, *Aspergillus niger*, *Cladosporium tenuissimum* and *Fusarium* was identified (28 cfu·cm^-2^,with a relative abundance of 6.70%).

Seven species of fungi were isolated from seeds stored for 60 d at 91% RH, and the total colony number was 106×10^4^ cfu·cm^-2^. The dominant microflora constituent on the seed surface was *Aspergillus jensenii* (relative abundance: 60%) at a density of 60×10^4^ cfu·cm^-2^ (Fig 3, A3), followed by *Aspergillus versicolor* (relative abundance: 75%) at a density of 22×10^4^ cfu·cm^-2^ and other microflora, including *Penicillium sp*, *Aspergillus aureus*, *Penicillium citrinum*, *Fusarium* sp. and *Aspergillus fumigatus*. Two species were isolated from the seed embryo, and the total colony number was 139×10^4^ cfu·cm^-2^. The dominant microflora constituent was *Penicillium* (relative abundance: 79.86%) at a density of 111×10^4^ cfu·cm^-2^ (Fig 3, A4), followed by *Aspergillus fumigatus* (relative abundance: 20.14%) at a density of 28×10^4^ cfu·cm^-2^. The above results showed that the RH of the storage environment could affect not only the species and distribution of the microflora on the seeds but also the population density.

### Vigor of seeds stored under different conditions

#### Seed germination

**Fig 4:**
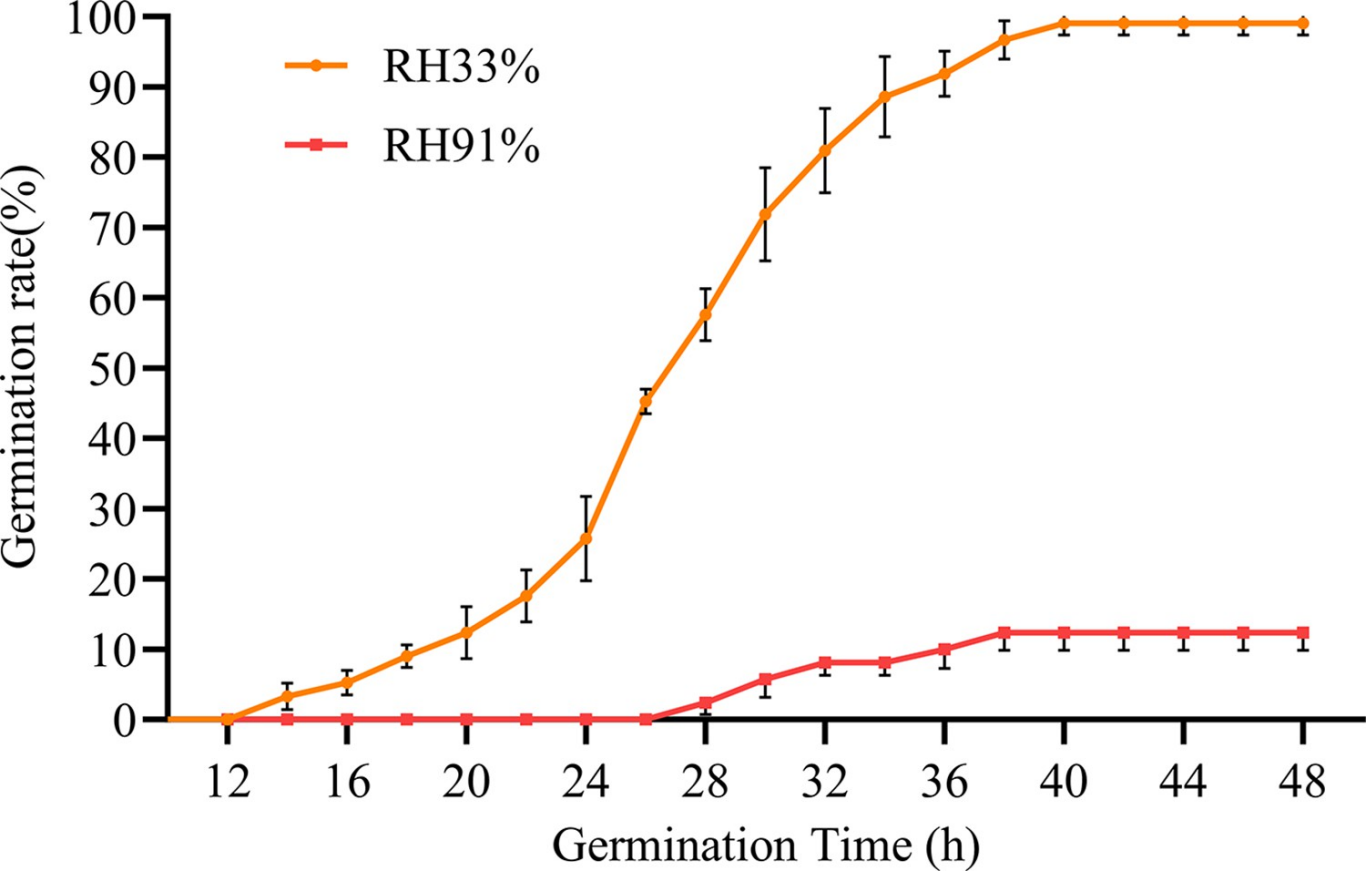
Seed germination rate curves under different storage conditions.

Seed germination is the main index used to evaluate seed vigor. The number of germinated seeds at different time points (expressed as the germination rate) was determined, and a germination curve was drawn (Fig 4). There were significant differences in the germination rates of maize seeds stored under different RHs. After the seeds were stored at room temperature (26 ± 2°C) and 33% RH for 60 d, there was no obvious effect on seed germination. The earliest germination time was 12 h after imbibition, the average germination time was 26 h when the germination rate reached 50%, and the average germination time was 42 h when the germination rate reached the highest point (99.05%). Most seeds germinated between 22 and 32 h after imbibition. The germination rate of the seeds stored at 91% RH decreased to 12.38%. These results showed that the RH of the storage environment strongly affects seed germination.

#### Seed respiration rate

**Fig 5:**
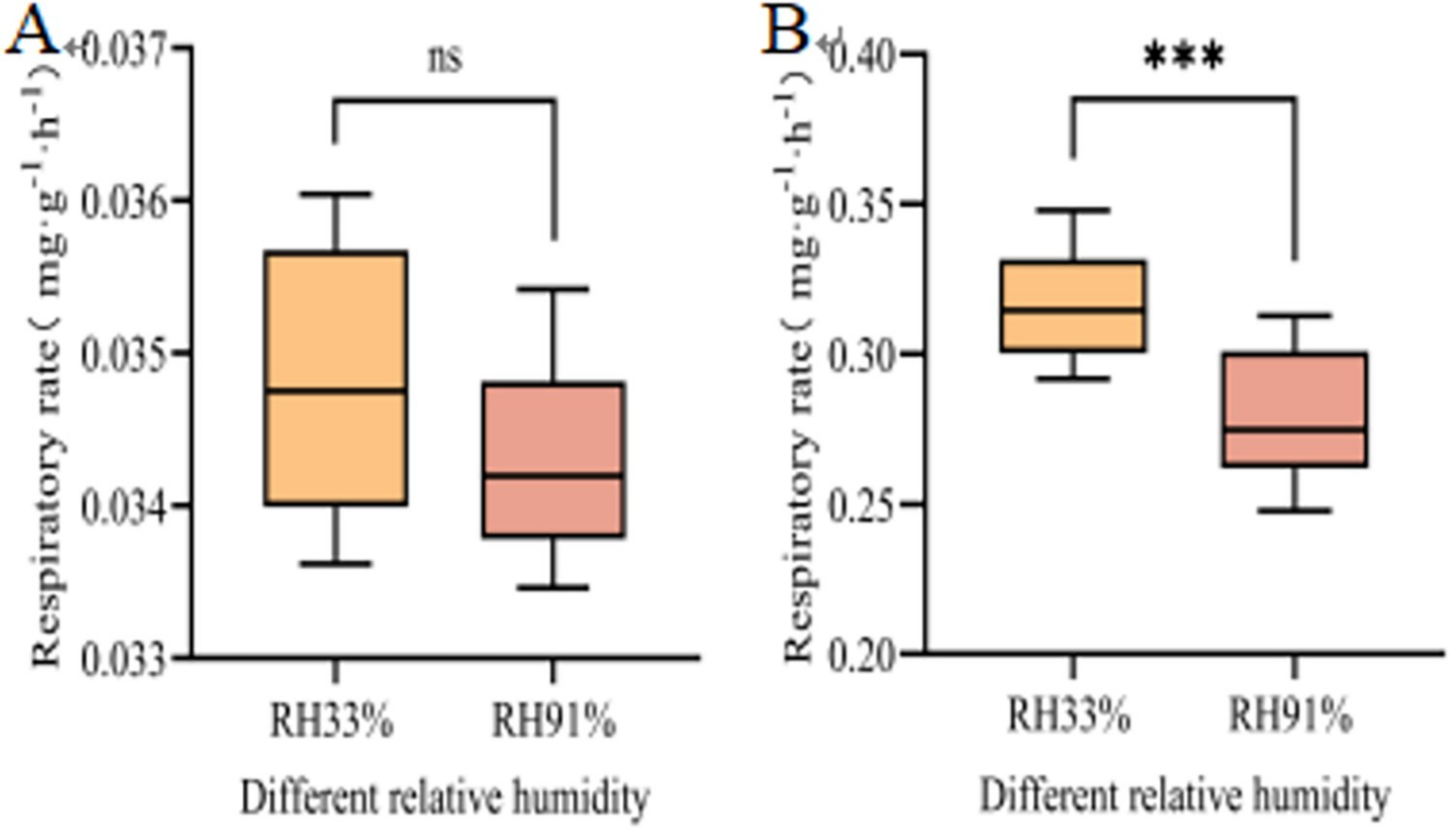
Changes in the respiration rates of seeds stored under different conditions. A and B represent the respiration rates of seeds at 0 h and 24 h after imbibition, respectively. Significant differences between the two treatment groups are indicated (***, P≤0.001 and ns, no significance), as determined by the independent Student’s t-test.

The seed respiration rate is not only an important index reflecting metabolic activity but also a specific manifestation of seed vitality. The respiration rates of seeds stored for 60 d under different RHs were determined (Fig 5). The respiration rates of the stored seeds were very low (0.0349 mg·g^-1^·h^-1^ and 0.0345 mg·g^-1^·h^-1^, respectively), and there was no significant difference before imbibition (Fig 5A). After imbibition for 24 h, the seed respiration rates significantly increased; however, there was a significant difference in the extent of the increase. The respiration rate of seeds stored at 33% RH was 0.32 mg·g^-1^·h^-1^, and the respiratory rate of seeds stored at 91% RH was 0.28 mg·g^-1^·h^-1^, a significant decrease of 0.04 mg·g^-1^·h^-1^ (Fig 5B). These results indicated that the RH of the storage environment strongly affects the degree of seed respiration.

#### Seed viability

**Fig 6:**
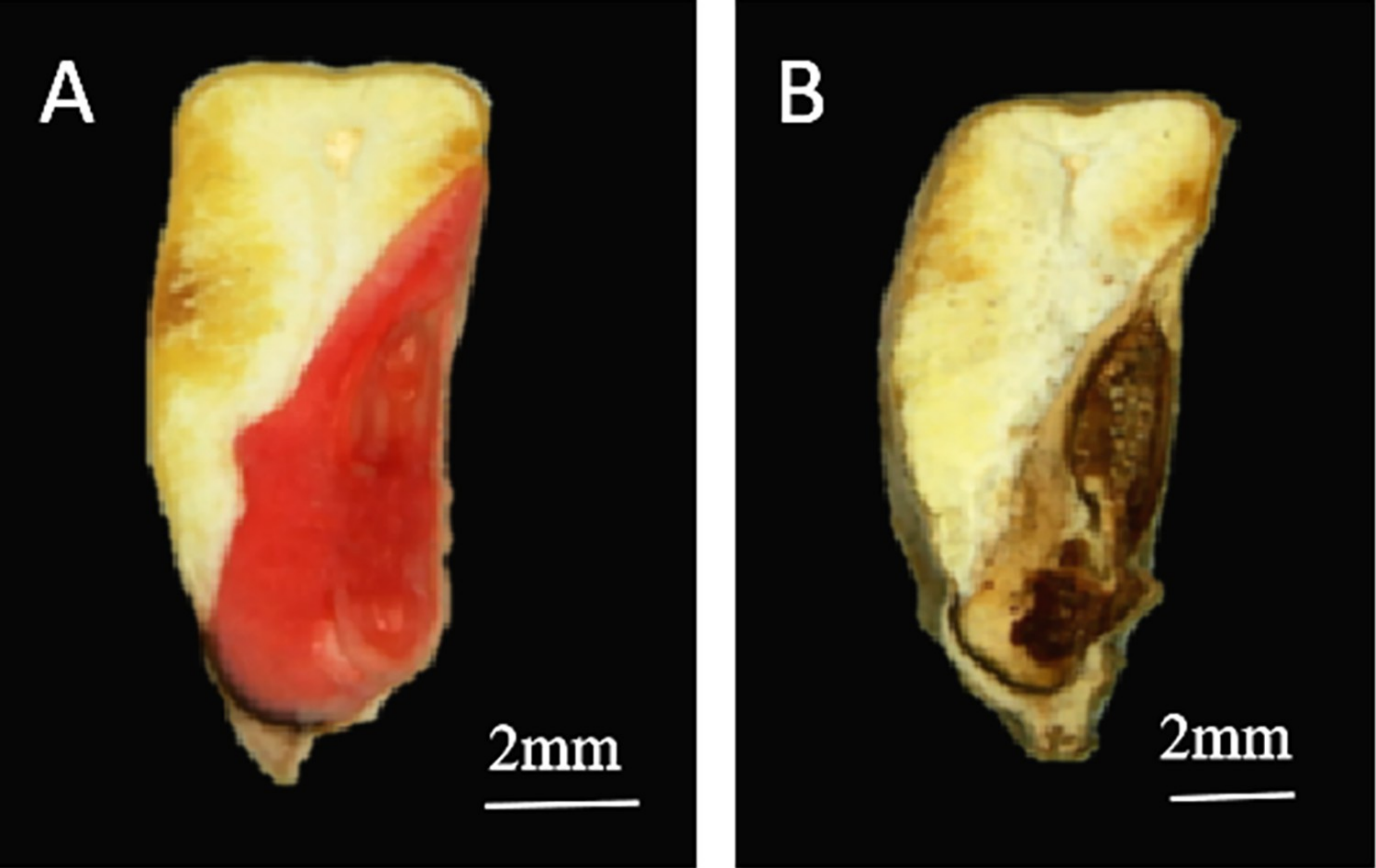
TTC staining of seeds stored under different environmental conditions. A and B represent seeds stored for 60 d at 33% RH and 91% RH, respectively. Scale bars: 2 mm.

Seed viability refers to the potential germination ability of seeds and the vitality of seed embryos, and seed viability can be checked via the TTC staining method. TTC staining of the seed embryos (Fig 6) revealed a significant difference in the viability of the seeds stored under different RHs (33% RH and 91% RH). The embryos of the seeds stored under 33% RH were intensely stained with TTC dye and appeared dark red (Fig 6A). These findings indicated that the embryos of the seeds were strongly viable. The seed embryos stored at 91% RH became brownish black and were difficult to stain with TTC dye (Fig 6B), which indicated that the seed embryos had lost viability. Whether the loss of viability of the seed embryo was directly related to the change in the microflora at the location of the seed embryo needs to be further examined.

#### Effects of different storage conditions on the MDA content of seeds

**Fig 7:**
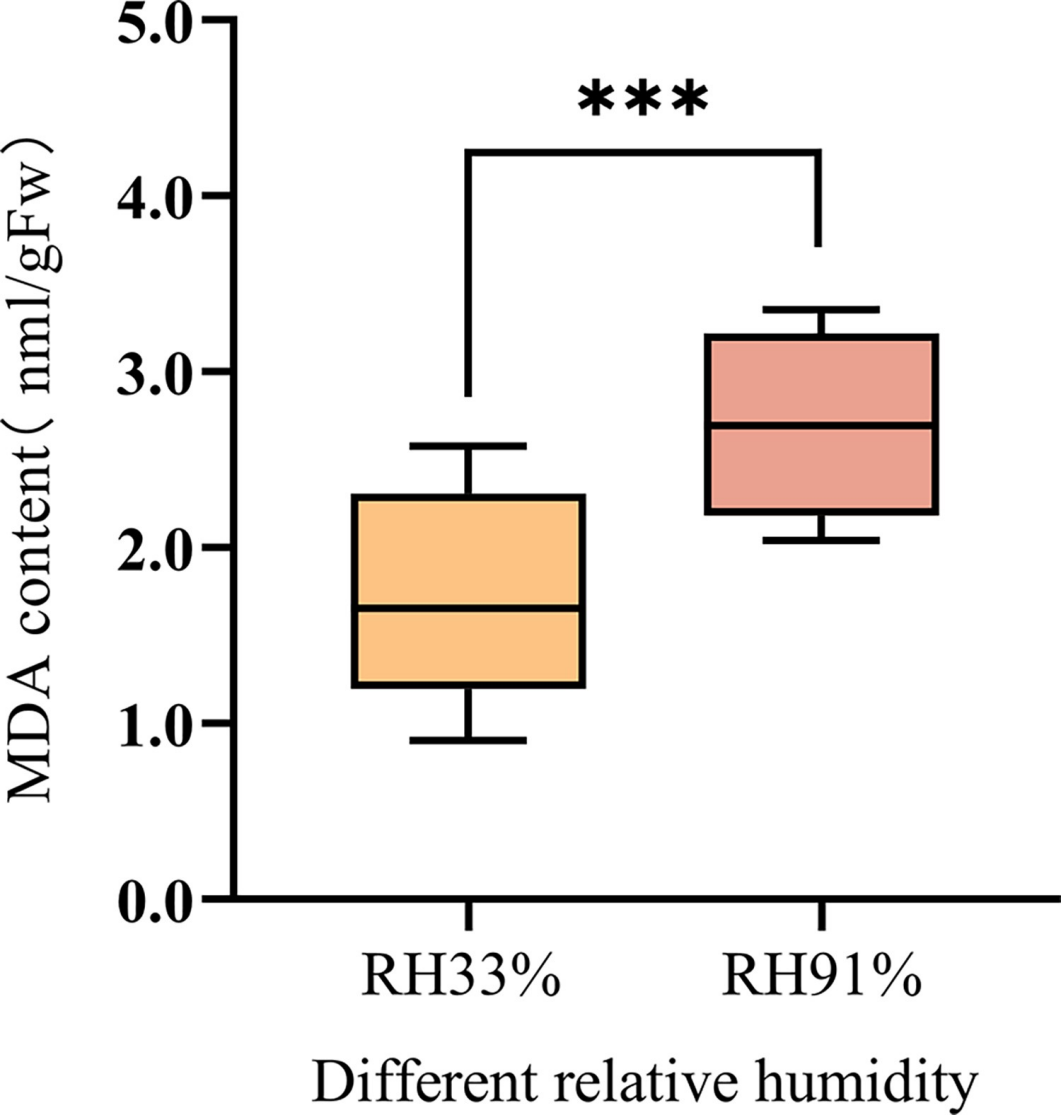
Changes in the MDA content of maize seeds stored under different conditions. Significant differences between the two treatment groups are indicated (***, P≤0.001), as determined by the independent Student’s t-test.

MDA is the final product of lipid peroxidation, and its content can be used as an index to evaluate the degree of damage caused by membrane oxidation. An increase in the MDA content indicates that the oxidative damage to the cell membrane is more severe. The seed MDA content significantly differed in maize seeds stored for 60 d under different RHs (33% RH and 91% RH) (Fig 7): the MDA content was 1.72 nmol·g^-1^ in seeds stored at 33% RH, whereas the MDA content significantly increased by 0.99 nmol·g^-1^ in seeds stored at 91% RH. This result indicated that the RH of the storage environment strongly affects the integrity of the seed cell membrane system.

#### ATP content in seed embryos

**Fig 8:**
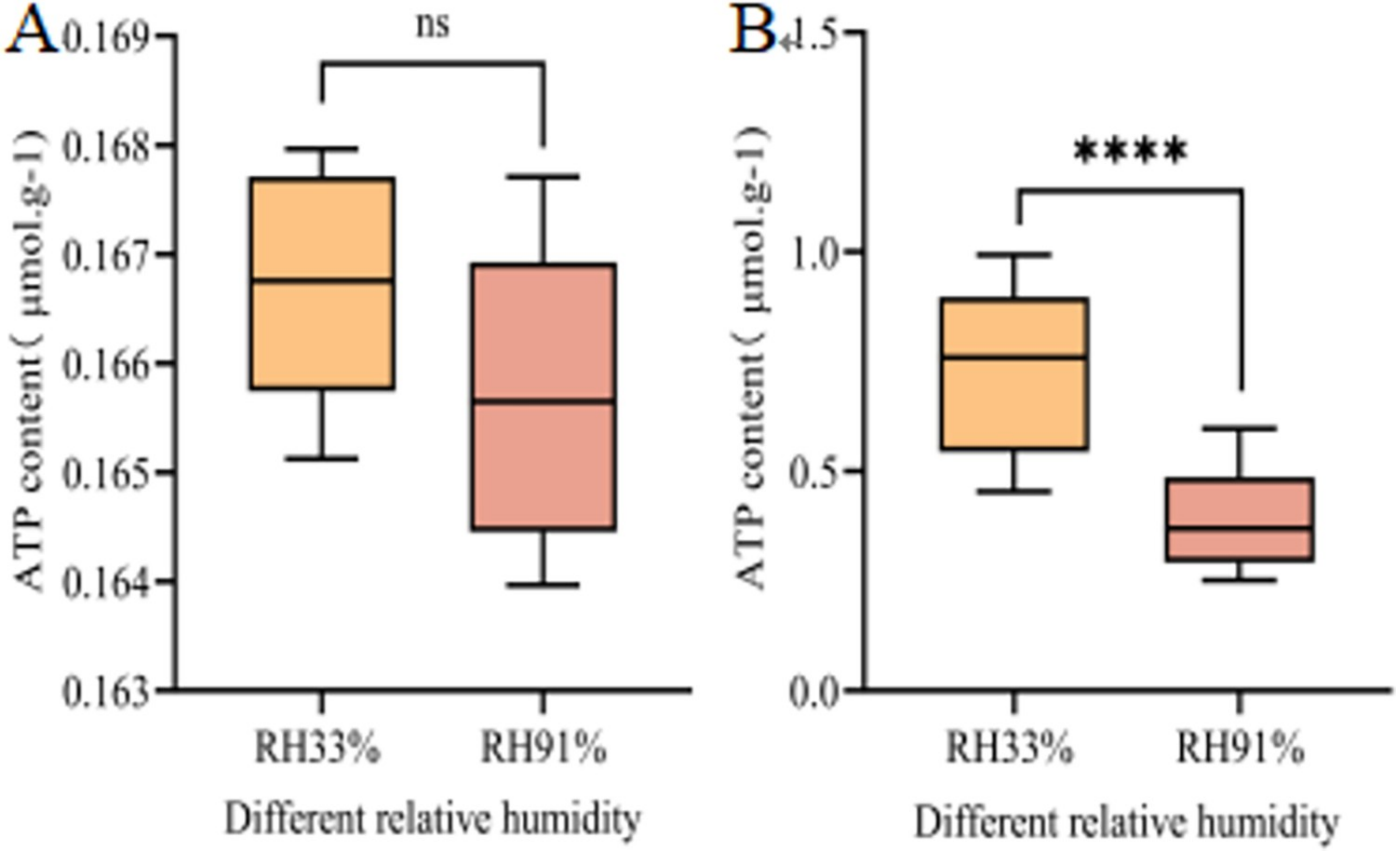
Changes in the ATP content of maize seed embryos under different storage conditions. A and B represent the ATP Content of seeds at 0 h and 24 h after imbibition, respectively. Significant differences between the two treatment groups are indicated (****, P≤0.0001 and ns, no significance), as determined by the independent Student’s t-test.

The ATP content, serving as a crucial indicator for assessing seed vigor serves as a direct source of energy for vital life processes that regulate seed germination and seedling establishment. ATP content determination revealed that the ATP content was very low in the seeds stored under different conditions before imbibition, and there was no significant difference between them (Fig 8A). After imbibition for 24 h, the ATP content of the seed embryo significantly increased. The ATP content in the seed embryos stored at 33% RH was 0.72 μmol·g^-1^, and in the seed embryos stored at 91% RH, the ATP content significantly decreased by 0.33 μmol·g^-1^ (Fig 8B). These results suggest that high-humidity storage conditions decrease ATP synthesis, resulting in an insufficient energy supply for the growth of seed embryos and ultimately reduced seed vigor.

#### Integrity of ATP synthase subunit mRNAs in seed embryos

**Fig 9:**
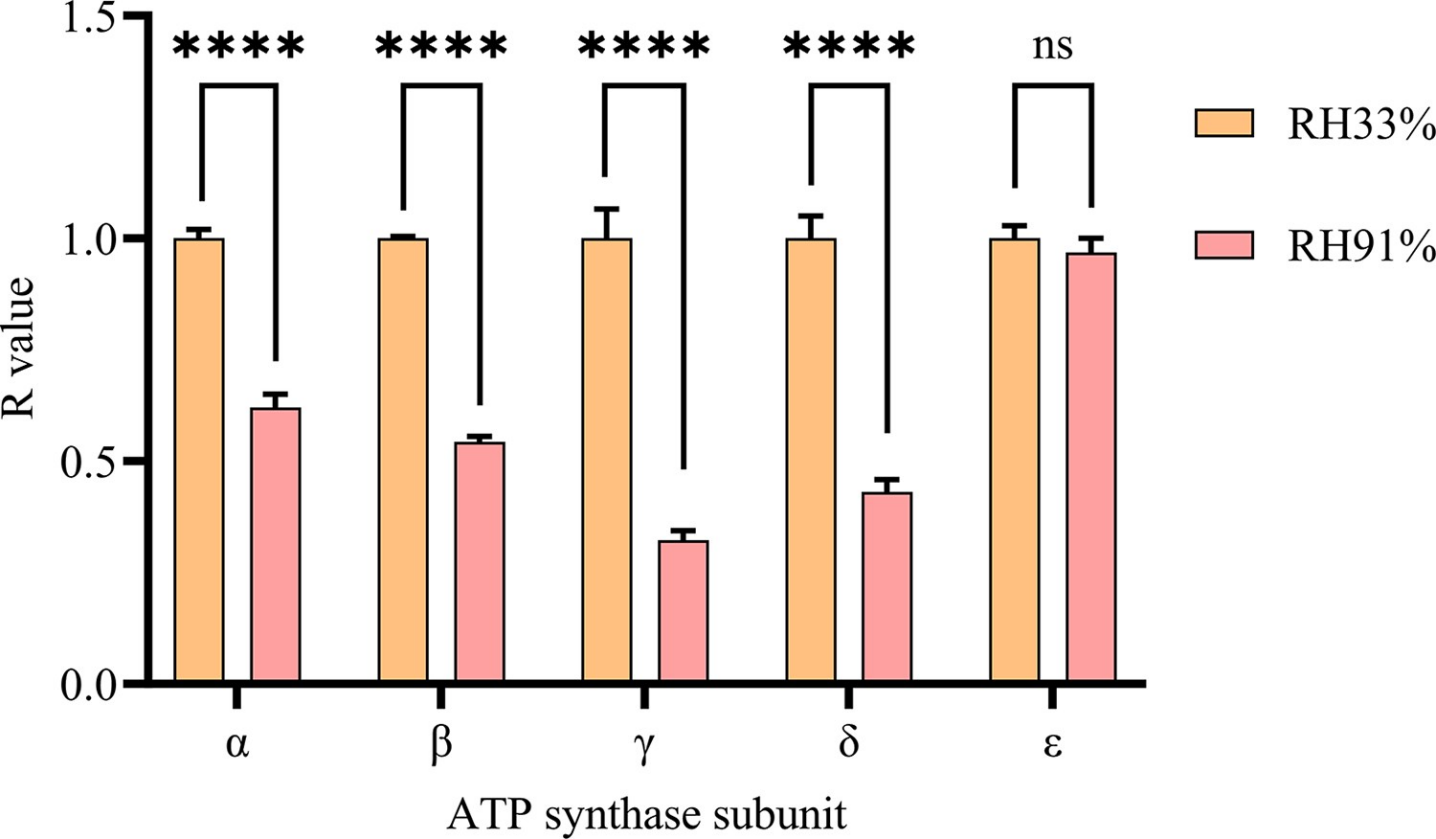
Changes in the mRNA integrity of ATP synthase subunits in maize seed embryos stored under different conditions. All data are expressed as mean±SD (n = 3). Significant differences between two treatment groups are indicated (****, P≤0.0001 and ns, no significance), as determined by the independent Student’s t-test.

The activity of ATP synthase is a key factor affecting ATP synthesis efficiency, and the quality of ATP synthase subunits can affect the activity of ATP synthase. The integrity of ATP synthase subunit mRNA in maize embryos stored under different conditions was analyzed via the reverse transcription blocking-double primer amplification method (Fig 9). The results demonstrated that the integrity (R value) of ATP synthase subunit α, β, γ and δ (except subunit ε) mRNAs in maize embryos stored at 91% RH decreased significantly, by 37.98%, 45.63%, 67.74% and 56.90%, respectively, compared with those of maize embryos stored at 33% RH. A decrease in mRNA integrity significantly impacts the function of ATP synthase subunits, thereby reducing ATP synthase activity, leading to decreased ATP synthesis efficiency and resulting in an insufficient energy supply for physiological activities such as seed embryo germination. Thus, impaired mRNA integrity of ATP synthase in the seed embryo during storage could be an important factor explaining the decrease in seed vigor.

## Discussion

### Changes in the seed microflora under different storage conditions

The environment is an important factor affecting the diversity of plant parasitic fungi, and the parasitic fungi present on seeds are quite different under different storage conditions [29]. The study conducted by Popovski and Celar [30] revealed that alterations in environmental conditions exerted an influence on the growth, reproduction, and fungal toxicity of species-transmitted fungi. Fungal spore formation and mold severity in grains are greatly influenced by temperature and relative humidity levels. Tonapi et al. [31] found a linear relationship between relative humidity levels and sorghum seed mold severity as well as fungal spore formation. Fungal spore formation and seed mold severity tended to increase as relative humidity levels increased from 95% to 98%. A change in seed moisture content increases the activity of parasitic microflora and further affects the abundance of the microflora [14]. Some mycotoxin-producing fungi, such as *Aspergillus*, *Fusarium*, *Alternaria* and *Penicillium*, can adapt to grow on low-moisture substrates but can also reproduce on stored seeds [32]. Huang [33] studied the mold activity of rice, corn, and wheat seeds stored at 25%, 75%, 85%, and 95% RH and reported that, under elevated RHs, the activity was 2% greater than that at a safe RH. The results revealed that the higher the RH was, the greater the microbial activity. Furthermore, the different storage conditions lead to great differences in the species and distribution of parasitic microorganisms in plants [34].

In this study, the microflora on the seed and embryo surfaces was notably affected by the storage conditions. Different dominant microflora constituents were isolated from the seeds stored at 33% and 91% RH, which indicated that the environmental RH could affect the type and number of microflora constituents on the seeds, which was consistent with previous research results. In addition, the number of colonies isolated from the whole seed surface with increasing ambient RH was much lower than the number of colonies from the seed embryo, which could be attributed to the nutrient-rich nature of the seed embryo, which is more suitable for the growth of microorganisms. This specific parasitization of the seed embryo accelerates the destruction of the seed embryo structure, which indirectly leads to a reduction in seed viability.

#### Seed vigor under different storage conditions

Environmental relative humidity is one of the important factors affecting seed storage vigor [35]. When the relative humidity is too high, the seeds absorb too much water, resulting in seed rot, mildew, and, consequently, reduced seed vitality [36]. Bakhtavar et al. [37] reported that increased seed water resulted in seed deterioration and reduced seed germination. Wang et al. [28] also reported that in a maize seed artificial deterioration experiment, a high-humidity storage environment led to a decrease in the seed germination rate, a decrease in germ viability, damage to ATP synthase mRNA, and a decrease in ATP content. The results of this study revealed that with increasing RH under different storage conditions, the seed germination rate decreased, the degree of TTC staining gradually decreased, the MDA content increased, the respiration rate decreased, ATP synthase subunit mRNA integrity decreased, and the ATP content decreased. These findings are the same as those of previous studies on the seed aging of soybean [38], cotton [39], maize [40] and wheat [41].

The survival, growth and pathogenicity of parasitic fungi on seeds are influenced by different conditions. The rapid propagation of saprophytic fungi such as *Rhizopus*, *Penicillium* or *Aspergillus* can lead to a decrease in corn grain storage quality, shrinkage, mildew and other problems [32, 42, 43]. In the process of fungal parasitism, a series of extracellular hydrolases, such as cellulase, amylase, pectinase and protease, are secreted to decompose seed nutrients (such as proteins, lipids and polysaccharides) [44], thus reducing the germination rate of seeds. Fungal parasites also secrete toxic metabolites, such as aflatoxins, which shorten the storage life of seeds [45]. Studies by Horbach et al. [46] have shown that fungi induce the production of reactive oxygen species (ROS) by secreting toxins or cell wall-degrading enzymes, thus killing the host. Alshannaq and Yu [47] reported that grain is a good substrate for microbial growth and that many toxic fungi, such as *Aspergillus*, *Penicillium*, *Curvularia*, and *Fusarium*, can infect corn plants and cause serious diseases, such as seed, root, stem, ear and grain rot. Nelson [48] reported that seed‒fungus interactions affect seed vigor. Halloin [49] reported that fungi can lead to seed deterioration during storage. Mackin et al. [50] confirmed that a direct relationship exists between fungal infection and a decrease in seed vigor in wheat. Meyer et al. [51] reported a decrease in the germination ability of *Bromus tectorum* L. seeds infected by fungi. In our work, the increase in RH in the storage environment not only changed the species and distribution of the microflora on the seeds of the Zhengdan958 cultivar but also caused severe decreases in or a total loss of seed vigor. Therefore, we suggest that the alteration of the microflora of the seed surface caused by the increase in RH in the seed storage environment is an important reason for the decrease in seed vigor.

## Conclusions

According to previous studies and the results of this research, we hypothesize that the species change and increased density of the microflora on the seed surface induced by an increase in RH in the storage environment is an important reason for the decrease in seed vigor. The mechanism of the decrease in seed vigor caused by the presence of different microflora constituents and their metabolic activities needs to be further studied.

## Supporting information

S1 Table Germination rate of maize seed under different storage conditions. (XLSX)

S2 Table Respiratory rate of maize seeds under different storage conditions. (XLSX)

S3 Table MDA content of maize seed embryos under different storage conditions. (XLSX)

S4 Table ATP content of maize seed embryos under different storage conditions. (XLSX)

S5 Table ATP synthase subunit qPCR of maize seed embryos under different storage conditions. (XLSX)

S6 Table Maize seed surface area under different storage conditions. (XLSX)

S1 Raw images (DOCX)
