# Supplementary figures and images for: Changes in the microflora on the seed surface and seed vigor of maize (Zea mays) under different conditions
Source: PLoS One. 2024 Nov 21;19(11):e0311258. doi: 10.1371/journal.pone.0311258 (PMC11581300; doi:10.1371/journal.pone.0311258)

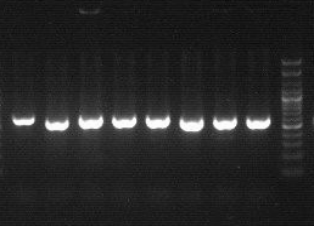


S7_raw_images

Supplement: S1 Raw images — (DOCX) [file pone.0311258.s007.docx]
